# New Progress on Fiber-Based Thermoelectric Materials: Performance, Device Structures and Applications

**DOI:** 10.3390/ma14216306

**Published:** 2021-10-22

**Authors:** Yanan Shen, Chunyang Wang, Xiao Yang, Jian Li, Rui Lu, Ruiyi Li, Lixin Zhang, Haisheng Chen, Xinghua Zheng, Ting Zhang

**Affiliations:** 1 Institute of Engineering Thermophysics, Chinese Academy of Sciences, Beijing 100190, China; shenyanan@iet.cn (Y.S.); wangchunyang@iet.cn (C.W.); yangxiao@iet.cn (X.Y.); lurui@iet.cn (R.L.); chen_hs@iet.cn (H.C.); 2 University of Chinese Academy of Sciences, Beijing 100049, China; 3 Nanjing Institute of Future Energy System, Nanjing 211135, China; lijian@njiet.cn (J.L.); liruiyi@njiet.cn (R.L.); zhanglixin@njiet.cn (L.Z.); 4 Innovation Academy for Light-duty Gas Turbine, Chinese Academy of Sciences, Beijing 100190, China

**Keywords:** thermoelectric fiber, flexible, performance, fabrication, structures, application

## Abstract

With the rapid development of wearable electronics, looking for flexible and wearable generators as their self-power systems has proved an extensive task. Fiber-based thermoelectric generators (FTEGs) are promising candidates for these self-powered systems that collect energy from the surrounding environment or human body to sustain wearable electronics. In this work, we overview performances and device structures of state-of-the-art fiber-based thermoelectric materials, including inorganic fibers (e.g., carbon fibers, oxide fibers, and semiconductor fibers), organic fibers, and hybrid fibers. Moreover, potential applications for related thermoelectric devices are discussed, and future developments in fiber-based thermoelectric materials are also briefly expected.

## 1. Introduction

With the development of science and technology and the progress of industrialization, the increasing demand for electricity, the excessive consumption of fossil energy, and serious environmental problems have attracted more attention in the world in recent years [1]. Therefore, researchers exploring the pattern of eco-friendly and sustainable energy utilization have invested extensive effort in overcoming the ever-growing energy crisis [2]. Thermoelectric material, which is a kind of energy material that can directly transfer thermal energy to electrical energy, is increasingly recognized as a major potential to contribute significantly to improving the environment and reducing greenhouse gas emissions without moving parts, moving fluids, noise, and pollution [3]. Owing to the explosive development of wearable electronics (such as smartwatches, smart glasses, internal pacemakers, and sports bracelets), most conventional generators have limited applications due to their heavy, bulky, and inflexible characteristics [4]. Researching flexible and wearable thermoelectric generators (TEGs) to provide energy to wearable electronics proves an extensive task [5].

The principle of fiber-based TEGs (FTEGs) is the thermoelectric effect (or Seebeck effect), which means temperature difference can generate thermoelectric voltage when it exists between both sides of FTEGs [5]. The maximum energy conversion efficiency of a thermoelectric device depends only on the temperature difference between hot and cold ends and the ZT value of the material. The dimensionless figure of merit ZT = σS^2^T/κ is determined by the performance of the thermoelectric material itself, where T is the average temperature, κ is the thermal conductivity, and σS^2^ is described as the power factor. In this formula, S is the Seebeck coefficient, which can be described as S = ΔV/ΔT. σ is the electrical conductivity, which can be expressed as σ = neμ, where μ is electron mobility, and n is carrier concentration. Thermal conductivity κ = κ_e_ + κ_l_ is composed of the electronic thermal conductivity and the lattice thermal conductivity κ [5,6,7,8,9]. Generally, a high ZT value indicates excellent thermoelectric performance, which means a high Seebeck coefficient S and electrical conductivity σ and a low thermal conductivity κ. However, it is very difficult to optimize multiple parameters simultaneously due to the interdependence of the three parameters [7,10]. Over the past few decades, numerous studies have focused on exploring a rational design to improve TE thermoelectric property, such as resonant doping, band convergence, and energy filtering effects to enhance the power factor, solid solutions, Nano structuring, and all-scale hierarchical structuring for reducing κ [1,6,9,11]. Additionally, it is worth noting that the mechanical properties, flexibility, stretchability, and toxicity of thermoelectric materials should also be considered, since the wearable TEGs can attach conformably onto the skin and prevent heat losses during energy transfer from the human body [3]. In general, the traditional thermoelectric devices have the characteristics of rigidity, gravity, and brittleness.

Generally, most conventional flexible thermoelectric (TE) devices are constructed based on bulk rigid TE elements, films with flexible, elastomer substrates, and flexible fabrics [5,8,9,12,13,14,15]. Although these structures of flexible TE devices have been studied extensively and have the advantages of flexible and light-weight properties, they only bend in one direction, with poor air permeability, two-dimensional planar structure, and low damage tolerance, which lead to a lack of essential wearable properties and restrict their commercial application in wearable TEGs [3,4,16]. Compared with films and bulks, fiber-based TEGs have a unique structure feature (such as a large length-to-diameter ratio and natural flexibility) with the capacity for weaving, knitting, and winding [16,17].

Thus, fiber-based TEGs (FTEGs) will be promising candidates for self-powered systems that collect energy from the temperature difference between natural sources and the human body to sustain wearable electronics [4]. In fact, not only does the performance of FTEGs depend on the type of materials, which affects thermoelectric conversion efficiency and flexibility, but also, it is determined by the structures, which affects the efficiency of heat collection and utilization as well as wearability. FTEG materials can be classified into the following categories: inorganic fibers (e.g., carbon fibers, oxide fibers, and semiconductor fibers), organic fibers, and inorganic/organic hybrid fibers [2]. Inorganic thermoelectric materials have outstanding thermoelectric performance compared to other materials, but they are rigid and costly. On the other hand, organic materials have unique flexibility, low density, and ease of processing. Based on their structures, FTEGs can be categorized into one-dimensional structures (1D) in yarn/filament/fiber forms, two-dimension structures (2D) with coated organic or inorganic TE materials onto fabrics, and three-dimensional (3D) structures, which are one-dimensional thermoelectric yarn/filament/fiber assembled into textiles by weaving and knitting [3,5,8].

Considering that the review on enhancing the performance of FTEGs is outdated, in this work, we overviewed the performances and device structures of state-of-the-art fiber-based thermoelectric materials. We exemplified the particulars of different materials and structures, such as preparation method, characteristics, power performance, evaluation, analysis, and potential applications. Finally, the current challenges and outlook for the future development of fiber-based thermoelectric materials were briefly discussed.

## 2. Inorganic TE Fiber

### 2.1. Semiconductor TE Fiber

Compared with carbon fiber, semiconductor fiber including Bi-Sb-Te/Se, SnSe, PbTe, and Ag_2_Te fiber exhibits better TE performance due to its narrow band gap, low thermal conductivity, and unique structure. Thus, semiconductor materials based on nanowires [18,19,20,21,22,23,24], hollow nanofibers, coated glass fibers, and core fibers have broad prospects in applications of miniature TE devices and wearable electronics.

#### 2.1.1. Bi-Sb-Te/Se

**Figure 1:**
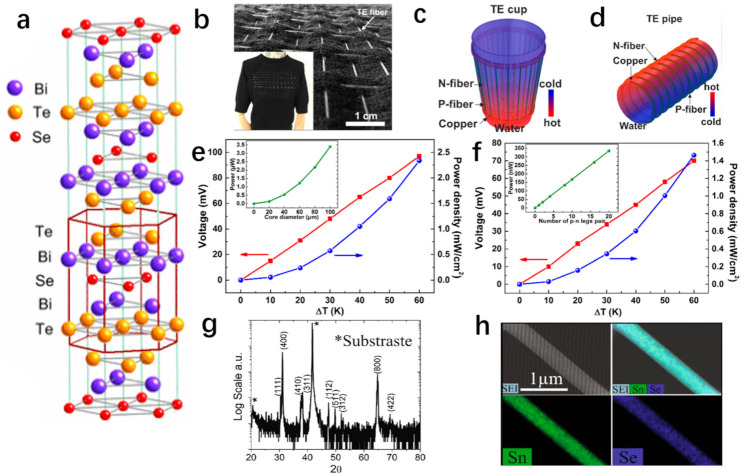
(**a**) Layered crystal structure of Bi_2_Te_2_Se showing the ordering of Te and Se atoms [31]. Copyright 2010 The American Physical Society. (**b**) The demonstration of a large surface wearable TE device, which is woven by TE fibers [29]. Copyright 2017 Elsevier. (**c**) The illustration of a TE cup that uses hot water to generate electricity [29]. Copyright 2017 Elsevier. (**d**) An illustration of a TE pipe which uses cold water to generate electricity [29]. Copyright 2017 Elsevier. (**e**) The function diagram shows the output voltage and power density of a TE cup covered with 7 pairs of TE fibers, which are represented by red line and blue line, respectively, within the temperature difference range of 0 to 60 K. The inside function diagram, simulated by finite element modelling (FEM), shows the relationship between the output power and the fiber core diameter at a temperature difference of 40 K [29]. Copyright 2017 Elsevier. (**f**) The function diagram shows the output voltage and power density of a TE pipe covered with 5 pairs of TE fibers, which are represented by red line and blue line, respectively, within the temperature difference range of 0 to 60 K. The inside function diagram, simulated by finite element modelling (FEM), shows the relationship between the output power and the fiber core diameter at a temperature difference of 40 K [29]. Copyright 2017 Elsevier. (**g**) X-ray diffraction pattern [19]. (**h**) Elemental mapping of SnSe nanowire (480 nm diameter) by scanning electron microscopy–energy dispersive X-ray spectrometry [19].

The semiconductors of group V–VI, exhibiting narrow band gaps, such as Bi_2_Te_3_-, Bi_2_Se_3_-, and Sb_2_Te_3_-based alloys, are widely used for TE devices with high ZT value within the temperature range of 200 to 400 K [18,25,26,27,28,29,30]. Bi_2_Te_3_, Bi_2_Se_3_, Sb_2_Te_3_, and their alloys crystallize in a rhombohedral structure belonging to the tetradymite space group $D_{3d}^{5}\left(R\bar{3}m\right)$, as shown in Figure 1a [31,32,33]. It was reported that a topological insulator (TI) of bismuth selenide (Bi_2_Se_3_) nanowires with uniform single-crystal structure successfully synthesized by stress-induced growth method [18]. The power factor of the 200 nm Bi_2_Se_3_ nanowire (NW) at room temperature was 39.2 × 10^−5^ Wm^−1^K^−2^, and the thermal conductivity κ, measured by 3ω technique, was 2.05 Wm^−1^K^−1^ [18]. At room temperature, the TE performance of Bi_2_Se_3_ NW rose to ZT = 0.06 [18]. To improve the preparation efficiency of one-dimensional TE materials, Bi_2_Te_3_ was put inside a glass tube and fabricated into Bi_2_Te_3_ core fibers via a modified thermal drawing process [27]. It was found that the Bi_2_Te_3_ unit cell had a hexagonal structure, and the angle between the hexagonal symmetry axis of the Bi_2_Te_3_ unit cell and the fiber axis was close to 90 degrees, which was beneficial to increase electrical conductivity [27]. There was also a phenomenon whereby a small amount of silicon and oxygen gradually and symmetrically diffused into the core, and the oxygen concentration was ~2 wt% [27]. The experimental result showed that the dimensionless figure of merit ZT is 0.73 at 300 K [27]. Similarly, Qian et al. fabricated Bi_2_Se_3_ core thermoelectric fibers using a thermal drawing method, and the 50 μm diameter Bi_2_Se_3_ core fibers exhibited an ultrahigh Seebeck coefficient of −150.85 μVK^−1^ and a figure of merit of 0.18 at a temperature of 300 K [28] Zhang et al. constructed the p-type Bi_0.5_Sb_1.5_Te_3_ core fibers and n-type Bi_2_Te_3_ TE core fibers into a two-dimensional fabric, as shown in Figure 1b [29]. To further demonstrate the ability of TE fibers to connect heat to cold sources at a distance effectively, a thermoelectric cup and a thermoelectric tube were manufactured as a thermoelectric generator, as illustrated in Figure 1c,d [29]. With a temperature difference of 60 K, the output voltage of TE cup and TE pipe reached 97 mV and 70 mV, and the power density achieved 2.34 mWcm^−2^ and 1.46 mWcm^−2^, respectively, as demonstrated in Figure 1e,f [29].

#### 2.1.2. SnSe

It is well known that SnSe has ultra-low intrinsic thermal conductivity and has an anisotropic thermoelectric property along a, b, and c axes due to its layered structure [34,35].

**Figure 2:**
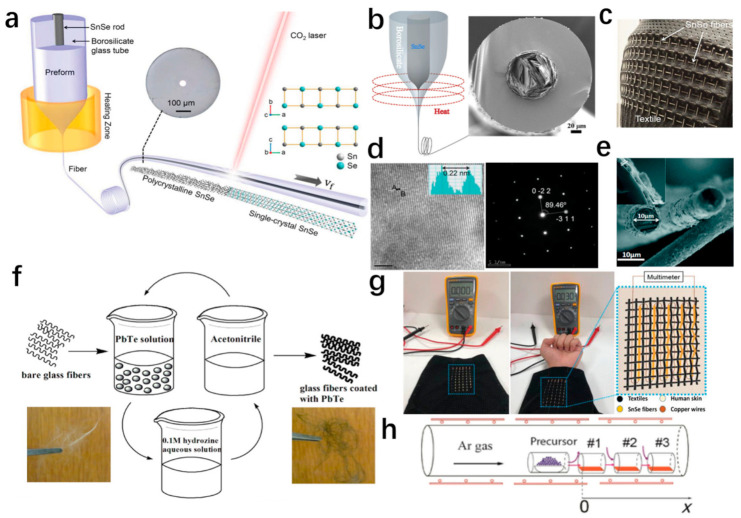
(**a**) The schematic shows the fiber drawing process of tin–selenium core fibers with glass cladding, and polycrystalline tin–selenium is converted into single–crystal tin–selenium using a laser. The inside illustrations show the microstructure of the cladding–core of tin–selenium fiber and the tin–selenium crystal structure $\mathrm{Fm}\bar{3}m$ along the c and b axis [38]. Copyright 2020 WILEY-VCH. (**b**) The illustration shows the thermal drawing process and the scanning electron microscope images of the cross-section of the tin–selenium fiber [35]. (**c**) A textile tin–selenium core fiber fabric with a large surface [38]. Copyright 2020 WILEY-VCH. (**d**) The image on the left is a high-resolution TEM (HRTEM) of single crystal tin–selenium. The diffraction pattern image of single-crystal tin–selenium using selected area electron diffraction (SAED) [38]. Copyright 2020 WILEY-VCH. (**e**) Scanning electron microscope image of PbTe nanocrystalline-coated fiber with a diameter and thickness of 10 μm and 300 nm, respectively [44]. Copyright 2012 American Chemical Society. (**f**) Scheme of covering glass fiber, which is coated with PbTe nanocrystals [44]. Copyright 2012 American Chemical Society. (**g**) The multidimensional tin–selenium fabric converts the temperature difference between the human body and the environment into electricity [38]. Copyright 2020 WILEY-VCH. (**h**) Schematic diagram shows the vapor transport reactor for growing PbTe and PbSnTe NWs [20]. Copyright 2016 Springer Nature.

In 2014, Zhao et al. indicated that the ZT for a single crystalline SnSe at 923 K reached 2.6 along axis b [36]. Moreover, Chang et al. showed an ultra-high ZT value of ~2.8 at 773 K in the out-of-plane n-type single crystal by modifying the temperature-dependent crystal and band structures deriving from the continuous phase transition [37]. In order to be applied to flexible and portable devices, SnSe nanowires and SnSe core fibers have been studied more and more extensively. For example, Hernandez et al. synthesized SnSe nanowires from a SnSe seed crystal by carrying out a catalyst-assisted thermal vapor–liquid–solid (VLS) process using Au as a catalyst [19]. X-ray diffraction pattern and SEM energy dispersive X-ray spectroscopy mapping (Figure 1g,h) indicate that the atomic ratio is close to 1:1, and the composition of Sn: Se is uniform without any crystalline SnO, SnO_2_, SnSe_2_, or Sn and Se by-products [19]. Thermoelectric parameters were measured at 370 K and exhibited a ZT value of 0.156 for the 130 nm diameter nanowire [19]. Furthermore, a SnSe core glass cladding fiber with ~94 μm core and ~220 μm cladding was prepared by a molten core drawing method, as shown in Figure 2b [35]. This method has potential to produce the next generation of low-cost, high-yield, one-dimensional TE fibers [35]. Nevertheless, the Seebeck coefficient of SnSe core reaches −151 μVK^−1^ at 300 K, the absolute value of which is a little less than that of a BiCl_3_ doping SnSe crystal (−176 μVK^−1^ at 300 K) due to the poly-crystal state core and the secondary phase SnSe_2_ [35]. Zhang et al. have combined the preform-to-fiber method with the laser heated base growth (LHPG) method to produce a single-crystalline fiber primarily focused on the realization of exceptional thermoelectric properties, as illustrated in Figure 2a [38]. In order to transform intrinsic polycrystalline SnSe into a single crystal with rock-salt $\mathrm{Fm}\bar{3}m$ phase, the CO_2_ laser was used to precisely control crystallization along the length of fibers, as shown in Figure 2d [38]. It follows that the ZT value of single-crystal SnSe reaches up to 1.94 at 862 K due to the existence of the rock-salt $\mathrm{Fm}\bar{3}m$ phase [38]. After several pairs of SnSe fibers were connected and woven into fabric (Figure 2c,g), the output voltage of 30 mV was generated at the temperature difference ~10K [38].

#### 2.1.3. PbTe

The phonon dispersion of rock-salt PbTe is similar to that of rock-salt SnSe [38,39]. PbTe is a considerable narrow gap TE material with a bandgap of 0.32 eV at 300 K, which can convert waste heat in the intermediate temperature zone of 500–900 K into electricity [40].

Moreover, PbTe hollow nanofibers with a wall thickness of about 20 nanometers were synthesized through a sequential process in three stages of electrospinning (synthesized silver nanofibers), electrodeposition (transformed into Ag_2_Te nanofibers), and cationic exchange reaction (from Ag^+^ cations to Pb^+^ cations) [40]. The synthesized 1D nanocomposite mats showed that the highest value of the Seebeck coefficient was 433 μVK^−1^ (at 300 K), when the content of remaining Ag was 30%. Additionally, the pure PbTe nanotubes realized the power factor as high as 0.567 μWm^−1^K^−2^ [40]. In addition, to enhance the thermoelectric properties, an alloying approach was combined with nanostructure to form unique topological surface states [20]. Figure 2h illustrates that Pb_1−x_Sn_x_Te nanowires were prepared with catalysts of Au nanoparticles via the vapor-transport approach [20]. The figure of merit ZT of the Pb_1−x_Sn_x_Te nanowires reaches ~0.035 for a width of 459 nm and thickness of 170 nm, and 0.018 for a width of 45 nm and thickness of 17 nm [20]. Additionally, using the chemical vapor transport method, single-crystalline PbTe nanowires were synthesized and exhibited Seebeck coefficients of −72 μVK^−1^ [21]. Besides chemical vapor transport method [21,41], PbTe nanowires can also be prepared by template-directed electrodeposition [42] and hydrothermal processing [43]. Furthermore, it was reported that PbTe-coated glass fibers were prepared using a scalable solution–phase deposition method to coat thermoelectric nanocrystals on the surface of flexible glass fibers (Figure 2f,e) and exhibited a power factor and ZT value of 0.41 μWm^−1^K^−2^ and 0.73 at 400 K, respectively [44].

#### 2.1.4. Ag_2_Te

Ag_2_Te forms the monoclinic phase (β) with a narrow bandgap of 0.06 eV at low temperature and transforms into the face-centered-cubic phase (α) with the carrier ions being Ag ions of more than 423 K [45,46,47]. Currently, many methods of preparing Ag_2_Te nanowires have been reported, including electrospinning [22], chemical vapor transport [23], solution-based synthesis route [48,49], microwave-assisted solution process [50], and solvothermal routes [51,52].

Ultralong Ag_x_Te_y_ hollow nanofibers were synthesized by combining electrospinning with a galvanic displacement reaction (GDR) to convert the electro-spun materials to the desired hollow chalcogenide nanofibers [22]. Low Ag-content Ag_x_Te_y_ nanofibers (<67%) were p-type, whereas high Ag-content Ag_x_Te_y_ nanofibers (>67%) were n-type [22]. The absolute number of the Seebeck coefficient decreased as the Ag content increased and exhibited the highest value of 589 μVK^−1^ in Ag_5_Te_95_ [22]. Additionally, high quality single-crystal Ag_2_Te nanowires were synthesized by a simple method of chemical vapor transport [23]. Ag_2_Te NWs can also be converted to films with excellent thermoelectric performance at room temperature using facile vacuum filtration and drop-coating methods [24]. Additionally, it is reported that the paper-based Ag_2_Te NWs film was made by transferring the film from the glass-fiber sheet to the copy paper via a new cold press method. The optimal power factor of paper-based Ag_2_Te NWs film was prepared at 30 MPa and displayed 192 μWm^−1^K^−2^ at a temperature of 468 K [53].

### 2.2. Carbon TE Fiber

Due to the special atomic arrangement of carbon materials, many allotropes of carbon exhibit high electrical and thermal conductivity properties [54,55]. However, the high thermal conductivity (>3000 Wm^−1^K^−1^) of carbon materials seriously limits its potential to be an excellent thermoelectric material, and various approaches of nanostructured defects (nanoribbons, nanopores, nanomeshes) [56,57,58] and physicochemical methods (doped Si atoms) [59] decrease its thermal conductivity. Carbon with nanostructures, such as carbon nanotubes and graphene, can become a candidate for thermoelectric materials, because the thermal conductivity is optimized by enhancing phonon–boundary scattering or phonon dispersions in nanostructures [54]. In previous reports, the thermal conductivity of multi-walled carbon nanotubes (MWCNTs) decreased from 2800 Wm^−1^K^−1^ to 500 Wm^−1^K^−1^, when the outer diameter increased from 10 nm to 28 nm [54,60,61]. The experiment results showed that the phonon and electron interactions between the multi-walled layers influenced thermal conductivity, which also increased with the reduction in the number of atomic walls in MWCNTs [60]. Additionally, the ZT value of zigzag graphene nanoribbons can be made as high as 4 at room temperature [60]. Using graphene ribbons with rough edges or introduced disorder, the graphene nanoribbon ZT was improved due to the strong suppression of thermal conductivity through phonon edge disordered scattering, while the electron transport did not deteriorate significantly [60].

#### 2.2.1. CNTs

CNTs are cylinders derived from rolling up a single layer of the graphite honeycomb lattice (called a graphene sheet), which lead to a special electronic band structure (1/3 of the nanotubes are metallic and 2/3 are semiconducting) [62]. In addition to high thermal conductivity and electrical conductivity, CNTs possess a one-dimensional structure, which contributes to a higher Seebeck coefficient and lower lattice thermal conductivity, as reported by Hick et al. [63,64]. This theoretical study demonstrated that a significant increase in ZT can be achieved with the decrease in structure from three-dimensional to one-dimensional, and the value of ZT is the highest in the one-dimensional structure [63]. This is mainly due to the change in the density of states and enhancing phonon scattering on the surface through reducing the dimensions of materials to limit the moving of phonons [63].

**Figure 3:**
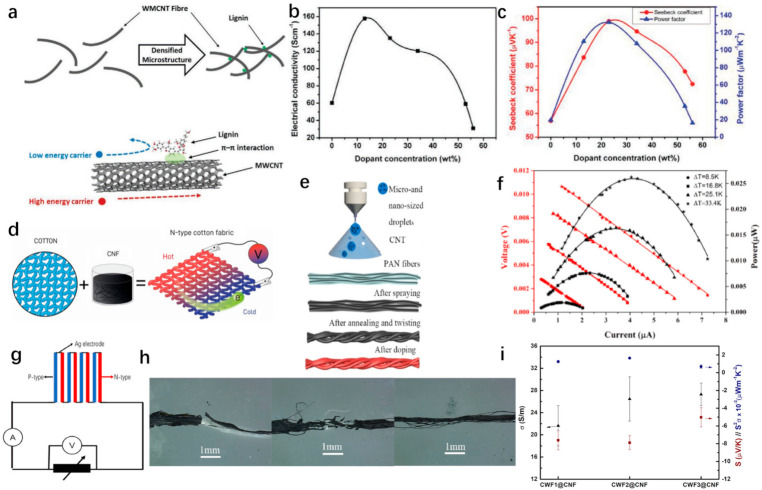
(**a**) MWCNT fiber and lignin form a dense microstructure and schematic diagrams of charge carrier filtering mechanism [68]. Copyright 2020 Wiley-VCH. (**b**) Electrical conductivity measured as a function of dopant concentration [68]. Copyright 2020 Wiley-VCH. (**c**) Seebeck coefficient and power factor measured as a function of dopant concentration [68]. Copyright 2020 Wiley-VCH. (**d**) Schematic diagrams of vapor grown N-type cotton fabric [73]. Copyright 2020, Springer Nature. (**e**) The fabrication process of CNTFs [70]. Copyright 2021 Elsevier. (**f**) Output power of TEG as a function under diverse temperature differences [70]. Copyright 2021 Elsevier. (**g**) The construction of thermoelectric device [70]. Copyright 2021 Elsevier. (**h**) CNTFs prepared by spraying CNTs slurry under 20 kV, 25 kV, 30 kV for 8 h, respectively [70]. Copyright 2021 Elsevier. (**i**) The measured electrical conductivity (black points), Seebeck coefficient (red points), and power factor (blue points) of dip-coated cotton fabrics [73]. Copyright 2020 Springer Nature.

Generally, fiber-based CNTs materials can be divided into p-type and n-type thermoelectric materials, depending on different categories of dopants, which determine the type of main charge carriers [65]. It was reported that CNTs became hole-doped in the presence of adsorbed oxygen due to oxygen’s electrons [66]. For single-wall carbon nanotubes, the one-dimensional SWNTs have large areas, strong covalent bonds between C-atoms in-plane, and the sensitivity of the π bonds out-of-plane. Therefore, these traits imply that solutions of redox molecules can easily dope in SWNT, which is strikingly different from the process by which defects are created by atomic substitution in conventional thermoelectric materials [64,67]. Furthermore, Culebras et al. prepared p-type carbon nanotube yarns (CNTYs) doped with lignin using impregnation, which was a sustainable and environmentally friendly material [68]. Densely interconnected nanostructures can be formed by the strong π–π interaction between lignin molecules and CNTY bundles, which can increase electrical conductivity and the Seebeck coefficient simultaneously (Figure 3a). Compared with the original CNTY sample, the conductivity and Seebeck coefficient are approximately doubled at a lignin content of 23 wt%, and the conductivity and Seebeck coefficient approximately doubled compared to the original CNTY sample. As a result, the power factor was significantly improved to 132.2 μWm^−1^K^−2^, more than six times that of the original CNTY, as illustrated in Figure 3b,c. In this work, 20 pairs of CNTY/lignin nanocomposite yarns effected a maximum output power of 3.8 W at a temperature gradient of 30 K [68].

It is worth noting that N-type CNTs are more challenging to obtain than P-type CNTs, because N-type CNTs should consider their doping quality and their stability in the air [65,69]. Figure 3e gives a schematic diagram of the CNTs fiber preparation process based on the electrostatic spray method, which deposits the CNTs on the insulating fibers with the micro-nano droplets [70]. With the applied electrostatic spraying voltage and time increasing, the surface of CNTs fiber became uniform and smooth, and the number of CNTs deposited on the fiber surface gradually increased, as shown in Figure 3h. The excellent adhesion between the CNTs and the insulating fibers contributes to superior mechanical properties, washing resistance, and reliability. In order to form n-type materials, the prepared CNTs fiber was doped with PEI dopants, and the Seebeck coefficient of N-type CNTs fiber remained unchanged after being exposed to air for 30 days, which indicated that it had good air stability [70]. In Figure 3f, four pairs of TE legs based on CNTF are connected with silver conductors to form a TEG. The device exhibited a maximum power of 26.2 nW with a typical output voltage of 6.46 mV and current of 4.06 μA at a temperature difference of 33.4 K and showed a power density of 0.20 nWK^−1^ per unit [70]. In addition, Ito et al. prepared CNTs fibers through the wet-spinning method and doped n-type CNTs with 1-butyl-3-methylimidazolium hexafluorophosphate ([BMIM]PF6) containing 10 wt% of dimethyl sulfoxide (DMSO). Eight pairs of fabricated π-type thermoelectric fabric (doped CNTs as n-type material, undoped CNTs as p-type material) exhibited an output power of 9 nW at a temperature difference of 25 K [71]. Additionally, by coating Nonionic waterborne polyurethane (NWPU)-based multi-wall CNTs thermoelectric composites on commercial polyester yarns, the fabric TEG generated an output power of 2.6 nW at ΔT = 66 K [72]. Unlike the n-type CNTs, which were fabricated with the utilization of any functionalization procedure and additives to prevent the inherent doping with oxygen, the dip-coated carbon nanofiber (CNF)-based cotton fabrics also showed n-type TE behavior [73]. Paleo et al. impregnated three different types of ordinary cotton fabrics with the same CNF-based ink dispersion and measured their thermoelectric properties (i.e., TEP, PF, and ZT) at room temperature (Figure 3f,g) [73]. Figure 3i shows that the cotton fabric with the largest linear density exhibits the best performance with negative thermoelectric power values around −8 μVK^−1^, a power factor of 1.65 × 10^−3^ μWm^−1^K^−2^, and a figure of merit of 1.14 × 10^−6^ [73]. In this study, the N-type characteristics of CNF can be interpreted as the compensated semi-metallic characteristics of CNF and the highly graphitic nature of their outer layers, which can cover the necessary graphite end plane and limit the grafting of oxygen functional groups to it.

Furthermore, the thermoelectric properties can be improved by forming CNTs/organic conductive polymers [74,75] or CNTs/ inorganic material [76] into composites. For example, the near room-temperature thermoelectric property of CNT yarn reaches to 1640–2160 μWm^−1^K^−2^ at a temperature range of 25–100 °C by making a composite with P3HT, followed by the F4TCNQ doping [74]. Wang et al. fabricated uniform core–shell nanostructured PEDOT/carbon nanotube (CNT) composites with the assistance of sodium dodecyl sulfate (SDS), and the nanostructure contributed to a high-power factor of up to 157 μWm^−1^K^−2^ at room temperature.

#### 2.2.2. Graphene

As a monolayer carbon atom in a honeycomb lattice, graphene exhibits a number of advantages over other materials, including high specific surface area, thermal conductivity, electrical conductivity, charge density, carrier mobility, and strength [55]. In previous reports and theoretical calculations, graphene has been confirmed to be a promising candidate for thermoelectric (TE) materials [55]. Ma et al. first reported experimental characterization of thermoelectric properties of graphene fiber in 2016 [77].

The graphene fibers had maximal a ZT value of 3.7 × 10^−6^ with increasing temperature from 100 to 290 K [77]. In order to improve the thermoelectric performance of graphene fibers, some strategies have been explored in recent years, such as halogen doping [78], polyethyleneimine ethoxylated (PEIE) doping [17], embedding CNTs, and doping with polyaniline (PANI) [79]. For example, as with halogen doping, bromine doping in graphene fiber could decrease the thermal conductivity by enhancing phonon scattering and increase the electrical conductivity and Seebeck coefficient by the Fermi level downshift [78]. Additionally, an integral p–n-connected all-graphene fiber was reported. P-type graphene fibers were treated with $N_{2}H_{4}H_{2}O$, and n-type graphene fibers were prepared via immersing into polyethyleneimine ethoxylated (PEIE) solution [17]. The fabricated p–n-type graphene fibers exhibited power factors of 0.78 μWm^−1^K^−2^ and 0.23 μWm^−1^K^−2^, and the thermal conductivity was measured as 0.26 Wm^−1^K^−1^ and 0.24 Wm^−1^K^−2^, from which the ZT was calculated as 2.04 × 10^−6^ and 0.96 × 10^−6^ [17]. Moreover, by mixing graphene and multi-wall carbon nanotubes (MWCNs), a composite material with 3D carbon-based hierarchical structure was fabricated, and then the composite material was introduced with PANI nano spacers to enhance thermoelectric properties [79]. Compared with the PANI fibers, carbon-based mixture blended with PANI fibers changed the type of thermoelectrical carrier from P-type to N-type [79].

### 2.3. Oxide TE Fiber

Compared with the elements in conventional intermetallic alloys, oxide TE materials are of low toxicity, cost-effective, and suitable for high-temperature applications due to their particular structure and excellent chemical stability [80,81,82,83]. Oxide TE materials include layered cobaltite (Ca_3_Co_4_O_9_, NaxCoO_2_) [81,84,85], SrTiO_3_ [86], CaMnO_3_, and ZnO. High electrical conductivity and low thermal conductivity are required to maximize and optimize ZT value in oxide TE materials. For oxide TE material, phonon transmission in the lattice contributes the predominant part of the total thermal conductivity [80]. For example, the thermal conductivity of NaCo_2_O_4_ crystal was measured to be 19 Wm^−1^K^−1^, in which phonons contributed 16.5 Wm^−1^K^−1^, and charge carriers contributed 2.5 Wm^−1^K^−1^ [81]. Therefore, enhancing phonon scattering, including applying nanostructures affecting the mean free path of phonons or introducing “impurity” atoms as scattering centers, is a strategy to reduce the thermal conductivity of lattice [80].

**Figure 4:**
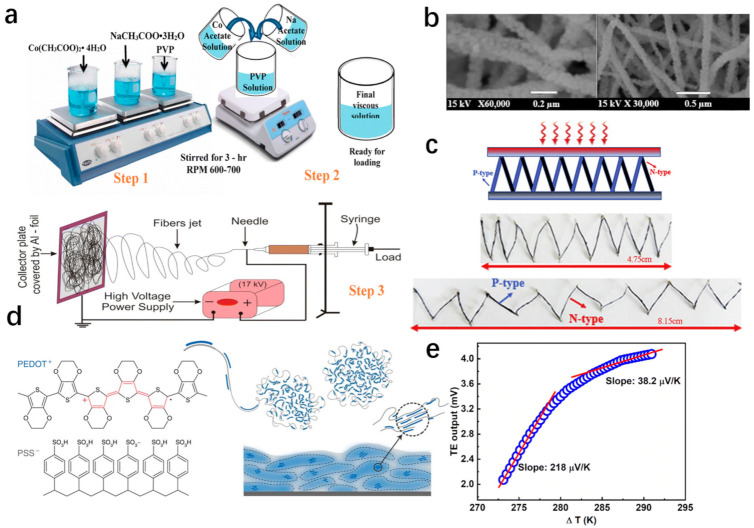
(**a**) The illustration for synthesis of NaCo_2_O_4_ nanofibers [84]. Copyright 2020 Springer Nature. (**b**) SEM micrographs of NaCo_2_O_4_ nanofibers calcined at 600 °C [84]. Copyright 2020 Springer Nature. (**c**) The schematic of fiber-based TE p–n pair [89]. Copyright 2019 Elsevier. (**d**) The Schematic diagram for the chemical structure of PEDOT: PSS and microstructure of the CP system [90]. (**e**) TE output voltage (∆V) vs. temperature gradient (∆T) [84]. Copyright 2020 Springer Nature.

Sodium cobalt oxide (NaCo_2_O_4_) is a transition metal, owing to a hexagonal layered structure in which CoO_2_ sheets are attached with the edge-sharing Na layers, CoO_2_ sheets tend to transport electrons, while Na layers decrease thermal conductivity [84]. Recently, Shah et al. synthesized PVP/NaCH_3_COO·3H_2_O/Co(CH_3_COO)_2_ nanofibers via an electrospinning technique and deposited the composite directly onto the silicon, as shown in Figure 4a. With a heat treatment of 600 °C, highly porous NaCo_2_O_4_ nanofibers with an average diameter of ~130 nm were obtained due to the decomposition of PVP and formation of γ-phase crystalline NaCo_2_O_4_ nanofibers (Figure 4b) [84]. The Seebeck coefficient was measured as 218 μVK^−1^ at a temperature gradient of 273–282 K and 38.2 μVK^−1^ near room temperature, as illustrated in Figure 4e, respectively. Additionally, Subramaniam et al. prepared tin oxide nanoparticles and vanadium oxide/tin oxide nanocomposites using a simple sol-gel method, and following that, they fabricated electrospun nanofibers by an electrospinning process [87]. Moreover, La_0.95_Sr_0.05_CoO_3_ nanofibers with diameters in the range of ~35 nm were also successfully prepared by the electrospinning process and exhibited a Seebeck coefficient value of 650 μVK^−1^ [86]. Compared with layered cobaltite fibers and perovskite fibers, which were fabricated by electrospinning and collected on rigid substrates, glass fiber coated with thermoelectric oxide can be used to make independent TE devices [88]. It is reported that the ZnO-coated glass fiber had better electrical conductivity than ZnO: Al-coated glass fiber, while ZnO: Al-coated fiber had a higher Seebeck coefficient of −246 μVK^−1^, which was −106.29 μVK^−1^ compared with ZnO-coated fiber [88].

## 3. Organic TE Fiber

In the past few decades, commonly used inorganic TE materials have been widely studied due to their high TE performance, while the disadvantages, such as high cost, rigidness, toxicity, and poor processability, limit their integration into wearable electronic devices [75,91]. In contrast, due to low cost, high flexibility, nontoxicity, and low thermal conductivity, conductive polymers, such as polyaniline (PANI), polypyrrole (PPy), polythiophene (PTh), and Poly(3,4-ethylenedioxythiophene): poly(styrenesulfon-ate) (PEDOT:PSS), have recieved more and more attention and have become one of the most promising candidates in flexible TEGs [91]. Generally, polyaniline (PANI), polypyrrole (PPy), and polythiophene (PTh) have been reported to have stable TE properties, but their ZT values were low compared with that of PEDOT:PSS. As a result, those conductive polymers with low ZT value can combine with carbon materials and inorganic materials to develop composite materials, which are potential solutions to practical applications of flexible TE devices. Additionally, hybrid materials are introduced in a later section.

PEDOT:PSS consists of positively charged conductive conjugated PEDOT and negatively charged insulating PSS and was first reported with a Seebeck coefficient of 12 μVK^−1^ in 2002 [92,93]. Figure 4d illustrates the chemical structure of PEDOT:PSS and the commonly described microstructure of the conductive polymers system [90]. As an organic TE material with significant potential, PEDOT:PSS has attracted tremendous research attention in wearable energy-harvesting devices due to its flexibility, nontoxicity, high electrical conductivity, and promising thermoelectric properties [92,94]. Over the past few years, PEDOT: PSS has been extensively studied, and a great deal of research has been performed in the field of wearable energy supply.

**Figure 5:**
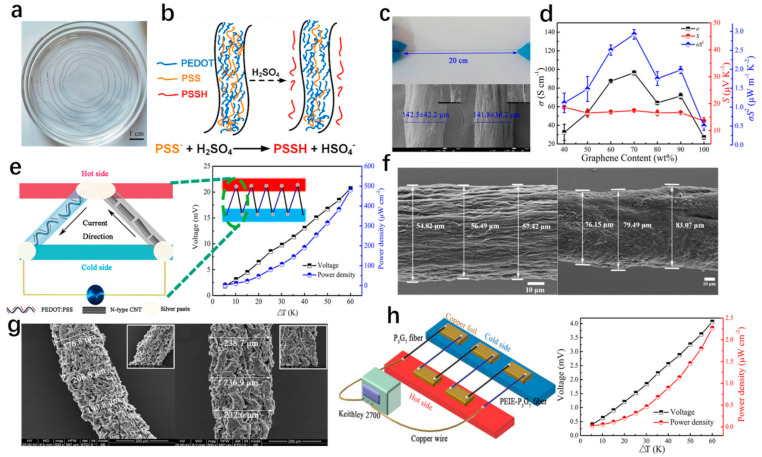
(**a**) Schematic diagram of coagulation bath, in which the PEDOT:PSS fiber is continuous spined [96]. Copyright 2020 Elsevier. (**b**) Schematic diagram of H_2_SO_4_ treatment to further enhance electrical and mechanical property [96]. Copyright 2020 Elsevier. (**c**) Digital photographs of as-fabricated PEDOT: PSS fiber and SEM images of PEDOT:PSS fibers before and after EG post-treatment [94]. Copyright 2018 American Chemical Society. (**d**) Thermoelectric performance of the graphene/PEDOT:PSS fiber [99]. Copyright 2020 American Chemical Society. (**e**) Schematic diagram for thermoelectric device consisting of five pairs of p−n legs (p-type PEDOT:PSS fibers and n-type CNT fibers), which are treated by EG, and the output voltage and power density as functions of temperature differences [94]. Copyright 2018 American Chemical Society. (**f**) SEM images of PEDOT:PSS/SWCNT0.6 and EG100-PEDOT:PSS/SWCNT0.6 [100]. Copyright 2020 Springer Nature. (**g**) Typical SEM images of P3G7 and PEIE-P3G7 hybrid fibers [99]. Copyright 2020 American Chemical Society. (**h**) The illustration of fiber measured device and relationship between output properties and temperature difference of the fiber device with three pairs of p–n legs [99]. Copyright 2020 American Chemical Society.

Recently, PEDOT:PSS cotton fabric was fabricated by impregnating cotton fabric with a polymer solution, achieving a Seebeck coefficient and power factor of 10.3 μVK^−^^1^ and 3.2 × 10^−3^ μWm^−1^K^−2^, respectively [95]. Similarly, Lan et al. obtained p-type TE fibers through the simple soaking of common cotton fabric (CNF) in PEDOT: PSS solution and n-type TE fibers by polyethyleneimine (PEI) treating p-type CNT with polyethyleneimine (PEI) [89]. The flexible fiber-based TE generators, assembled by connecting p-type PEDOT: PSS-coated CNF and n-type CNT fiber, consisted of eight pairs of p–n with an individual length of 0.8 cm, as shown in Figure 4c [89]. The generator produced an output voltage of 45.2 mV and output power of 375 μW at a temperature difference of 60 K [89]. The PEDOT:PSS fibers, prepared by a continuous wet-spinning process, were treated with sulfuric acid, which had effective modulation on composition and chain conformation, as shown in Figure 5a,b [96]. Under optimal conditions, PEDOT: PSS fibers delivered a Seebeck coefficient of 19.2 μVK^−1^ and a power factor of 147.8 μWm^−1^K^−2^ [96]. Five pairs of p-type PEDOT: PSS fibers and n-type Ni wires, attached on a flexible Kapton tape, generated a maximum output power density of 1.79 μWcm^−2^ and an output voltage of 4.87 mV [96]. In the same way, Sarabia-Riquelme et al. reported that sulfuric acid drawing improved the PEDOT: PSS fiber properties, resulting in a value of electrical conductivity of 4039 ± 215 Scm^−1^, Young’s modulus of 22 GPa, and break stress of about 550 MPa [97]. Kim et al. improved TE performance and mechanical properties by dedoping polyethyleneimine (PEI) after wet-spinning PEDOT:PSS fibers into sulfuric acid [98]. Moreover, a highly conductive p-type PEDOT:PSS fiber was produced by gelation process with certain addition of sulfuric acid and took a post-treatment with ethylene glycol (EG) and dimethyl sulfoxide (DMSO), as shown in Figure 5c [94]. Based on the experimental results, the optimal power factor for PEDOT: PSS hydrogel fiber, treated with ethylene glycol (EG) and dimethyl sulfoxide (DMSO), reached 4.77 μWm^−1^K^−2^ and 3.79 μWm^−1^K^−2^, respectively [94]. A TEG was assembled with p-type PEDOT: PSS fibers and n-type CNT fibers and exhibited an output voltage of 20.69 mV and an output power density of 481.17 μWm^−1^K^−2^ at a temperature difference of 60 K, as shown in Figure 5e [94].

## 4. Hybrid Fiber

### 4.1. CNT/Organic Fibers

It is well known that organic TE materials, such as PANI, PEDOT:PSS, and poly(N-vinylpyrrolidone) (PVP), are proved highly attractive due to low thermal conductivity, easy processing, and excellent flexibility, while their low electrical conductivity and Seebeck coefficient limit improve TE properties [101]. Recently, incorporating carbon nanomaterials such as CNT and graphene into organic TE materials has led to an energy-filtering effect between the interface between organic materials and carbon nanomaterials, which optimizes electrical/thermal transport behaviors and enhances the TE performance [101]. Furthermore, the introduction of carbon nanomaterials can also increase Young’s modulus of organic materials [100]. Thus, hybrids of carbon nanomaterials with organic TE materials are one of the potential methods of improving the performance of organic thermoelectric fibers.

PEDOT: PSS/single-walled carbon nanotubes (SWCNTs) hybrid fibers were prepared by a gelation method [100]. With the introduction of SWCNTs, Young’s modulus of the hybrid fiber was increased to 1.6 GPa when the weight content of SWCNTs reached 20% [100]. Subsequently, Figure 5f shows a change in the surface composite after treating hybrid fibers with ethylene glycol (EG), and the power factor was promoted to 5.57 μWm^−1^K^−2^, due to the separation of the insulating PSS chains from the highly conductive PEDOT chains [100]. To further improve the thermoelectric performance, core–shell nanostructured PEDOT/CNT composites were successfully synthesized via a facile in situ chemical solution polymerization method and led to a high-power factor of 157 μWm^−1^K^−2^ [75]. A thermoelectric yarn-based generator was fabricated by coating PEDOT: PSS/CNT composites on cellulose yarn and output voltage of 1.5–2 mV at the temperature between the human body and ambient temperature [75]. In order to enhance the adhesion between conductive polymer and fabric substrate, Serrano-Claumarchirant et al. prepared coating fabrics with multiple-wall carbon nanotubes through layer-by-layer and by electrochemical polymerization of PEDOT, exhibiting a Seebeck coefficient of 14.3 μVK^−1^ [102]. Additionally, CNT/PEDOT: PSS composite fibers can also be prepared by a wet-spinning process with p-type and n-type power factors of 83.2 μWm^−1^K^−2^ and 113 μWm^−1^K^−2^, respectively. For other conductive polymers, they can also be used to form hybrid materials with CNT. Ryan et al. reported air-stable and flexible n-type yarns by coating MWCNTs and poly(N-vinylpyrrolidone) (PVP) nanocomposites on commercial sewing threads [103]. In order to further improve the wear and water resistance of coated yarns, a polystyrene-b-polyisoprene-b-polystyrene block copolymer (SIS) was provided as a protection layer [103]. In addition to conductive polymers, poly (vinylidene fluoride) (PVDF), an electrically insulating polymer, can form a composite with carbon fiber, and this composite possesses high stability of the power factors under ambient conditions. Kim et al. prepared p-type SWCNT/PVDF composite fibers using a simple wet-spinning method and n-type composite fibers with the addition of a PEI treatment [104]. In order to avoid PVDF on the influence of electrical conductivity, the average inter-bundle distance was reduced significantly using evaporation to prepare the pastes before wet-spinning [104]. As a result, the power factor of the p- and n-type SWCNT/PVDF composite fibers reached 378 ± 56 μWm^−1^K^−2^ and 289 ± 98 μWm^−1^K^−2^, respectively.

### 4.2. Graphene/Organic Fibers

In addition to CNT, graphene is a highly conductive filler based on theoretical calculations and previous experimental results. The hybrid fibers graphene and PEDOT: PSS were prepared by using a hydrothermal process with ascorbic acid (AA) in a tube mold, and n-type fibers were achieved by treating hybrid fibers with polyethyleneimine ethoxylated (PEIE), as shown in Figure 5g [99]. Compared with graphene fiber, the electrical conductivity of hybrid fibers significantly increased from 27.1 Scm^−1^ to 96.3 Scm^−1^ due to an increase in σ, which might be ascribed to the π−π stacking of graphene and PEDOT chains, and the power factor was about 2.9 μWm^−1^K^−2^, as shown in Figure 5g [99]. Figure 5h illustrates a fiber device with three pairs of p–n legs; the device output performances were 4.07 mV and 2.27 μWcm^−2^ [99]. In addition, polymeric TE composites were fabricated by grafting polypyrrole (PPy) onto the cheap graphene of reduced graphene oxide (rGO) in the bundled micro-tunnel of towel gourd sponge (TS) fibers [105]. After curing the polymeric TE composites with polydimethylsiloxane (PDMS), the interfacial energy barrier prevented phonon transport along the micro-tunnel and low-energy carrier transport across the interfaces. As a result, the PDMS/TS-rGO-PPy exhibited a high Seebeck coefficient of 84.2 μVK^−1^ and low thermal conductivity of 0.249 Wm^−1^K^−1^ [105]. Furthermore, El-Basaty et al. synthesized nanocomposites using PANI fiber and C-Mix (graphene–MWCNTs composite material), and Wang et al. synthesized graphene quantum dots (GQD)/ polydopamine (PDA)/PANI, CNT/PDA/PANI, and graphene nanosheets (GNS)/PDA/PANI ternary composites [79,106].

### 4.3. Non-Carbon Inorganic/Organic Hybrid Fibers

**Figure 6:**
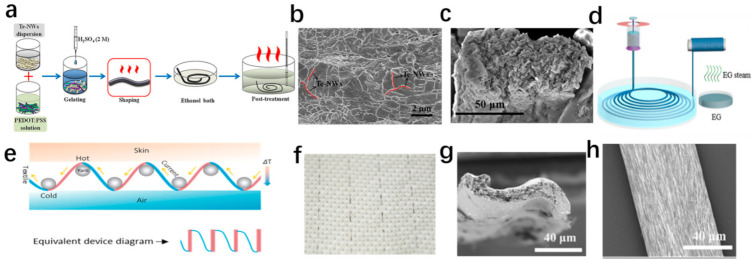
(**a**) The preparation and post-treatment of PEDOT: PSS/Te NWs hybrid fibers [107]. Copyright 2020 Elsevier. (**b**) PEDOT: PSS/Te NWs hybrid fiber (the content of Te NWs is 50 wt%) [107]. Copyright 2020 Elsevier. (**c**) Cross-section SEM image of PEDOT: PSS/Te NWs hybrid fiber (the content of Te NWs is 50 wt%) [107]. Copyright 2020 Elsevier. (**d**) Schematic illustration of the wet-spinning processes [16]. Copyright 2020, American Chemical Society. (**e**) Schematic illustration of working principle of TE fabric (blue—PEDOT: PSS/Te, brown—Ag) [16]. Copyright 2020, American Chemical Society. (**f**) Photos of TE fabric [16]. Copyright 2020, American Chemical Society. (**g**) SEM images of PEDOT: PSS/Te NW composite fibers cross-section [16]. Copyright 2020, American Chemical Society. (**h**) Surface SEM images of PEDOT: PSS/Te NW composite fibers [16]. Copyright 2020, American Chemical Society.

Non-carbon inorganic materials usually exhibit excellent TE performance. However, their toxicity, high cost, poor processing, and low flexibility also need to be solved [107]. PEDOT: PSS/Te NWs hybrid fibers were successfully prepared by gelation with facile post-treatment, as shown in Figure 6a [107]. Figure 6b,c present SEM images of PEDOT: PSS/Te NWs hybrid fiber (the content of Te NWs is 50 wt%) and its cross-section [107]. With the content of Te NWs increasing to 50 wt%, the Seebeck coefficient gradually increased from 18.3 to 27.2 μVK^−1^, and the electrical conductivity reached a maximum of 102.2 Scm^−1^ due to the more ordered PEDOT layer, which was led by the interaction between Te and PEDOT:PSS [107]. A simple fiber-based TE generator with six pairs of p–n legs (p-type PEDOT:PSS/Te fibers and n-type CNFs) was assembled and achieved a comparable output voltage and power density of 4.7 mV and 62.3 μWcm^−2^, respectively [107]. In Figure 6d, Xu et al. fabricated a composite fiber based on PEDOT:PSS/Te NWs by a facile wet-spinning process with a high-power factor of 78.1 μWm^−1^K^−2^; this holds the potential to scale up [16]. Figure 6g,h show the cross-sectional and surface SEM images of PEDOT: PSS/Te NWs composite fiber with Te NWs content of 50 wt% [16].

Moreover, a fiber-based TE device was designed by weaving TE fibers into a fabric, which achieved a mass specific power of 9.48 μWg^−1^, as shown in Figure 6e,f [16]. Additionally, a multistep dip-coating process and electrostatic interactions were used to fabricate Ag NW/PEDOT: PSS-coated yarn [108]. The Ag nanowire/PEDOT: PSS-coated yarns exhibited a high conductivity of ~320 Scm^−1^ and excellent robustness after washing, which was attributed to a strong electrostatic interaction between the PEDOT: PSS coating and the silk [108]. In addition, PAN and NaCo_2_O_4_ can be fabricated into composite nanofiber webs by dual electrospinning [109]. At a fabrication temperature of 700 °C, the Seebeck coefficient of composite nanofiber web increased from ~26.77 to ~73.28 μVK^−1^ with the increase in relative content of nanofibers [109]. Furthermore, PEDOT: PSS/Te NW/CuTe nanocomposite [110], Sb_2_Te_3_/PAN yarn [111], and other hybrid fibers were researched to explore a high-performance TE fiber.

## 5. Application

### 5.1. Sensor

**Figure 7:**
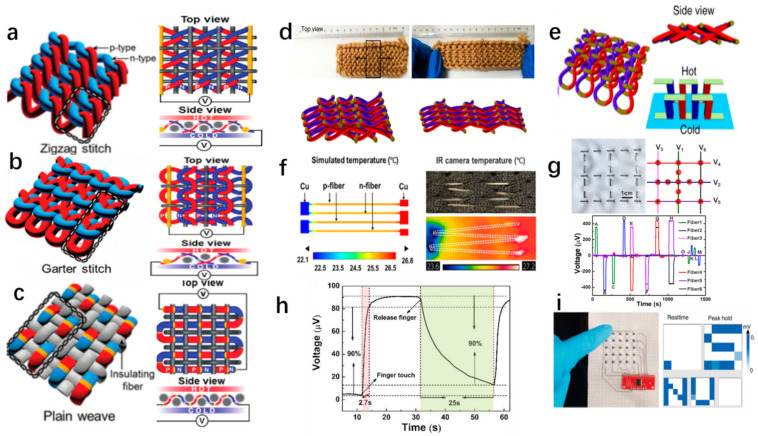
(**a**) A zigzag stitch of thermoelectric textiles [111]. Copyright 2016 WILEY-VCH. (**b**) A garter stitch of thermoelectric textiles [111]. Copyright 2016 WILEY-VCH. (**c**) A plain stitch of thermoelectric textiles [111]. Copyright 2016 WILEY-VCH. (**d**) Photographs of the top view and schematic after longitudinal stretching by 0% and 40%. The highlight area in a indicates the effective TE modules with 15 (3 × 5) units, the strain of which is equal to the overall textile [116]. (**e**) Illustration of three-dimensional out-of-plane TEGs without substrate by using interlocked in 2D space [116]. (**f**) Photographs of the FEM simulation and actual IR camera captured temperature profiles for the wearable cooling fabric with an input current of 2 mA [29]. Copyright 2017 Elsevier. (**g**) A 3 × 3 TE fiber array woven into a flexible fabric and the finger touching points schematically marked by capital letters. Moreover, the corresponding output voltages of the thermal sensor network for different thermal source positions when finger touches the marked points [113]. Copyright 2019 American Chemical Society. (**h**) Response time of a single TE fiber [115]. (**i**) Photograph of a 5 × 5 pixel touch panel with cross-stitched TE fibers, and photo of a “NUS” writing on the touch panel [13].

The temperature sensor is an important device applied to industrial production, medical diagnosis, and military defense and provides essential information about the dynamics of many physical, chemical, and biological phenomena [112,113,114]. To further meet the flexibility requirements, fiber-based temperature sensors are fabricated to be woven into fabrics or applied to curved surfaces [113]. Zhang et al. demonstrated a thermoelectric fiber consisting of semiconductor core and polymer cladding that operated in a wide temperature range with high flexibility, detection sensitivity, and accuracy [113]. A 3 × 3 thermal sensor array was assembled by six TE fibers with a length of 8 cm [113]. When the finger touched different points, the thermal sensor array achieved a quadrant location of the thermal source by the sign of the different voltage, as shown in Figure 7g [113]. In addition, Ruan et al. presented a figure self-powered flexible sensor based on the hollow PEDOT:PSS fibers, which were encapsulated on a low thermal conductivity polydimethylsiloxane (PDMS) layer [115]. Additionally, the flexible sensor achieved a highly selective response of about 2.7 s and provided a discriminant output voltage between human skin and the environment, as shown in Figure 7h [115]. Additionally, a touch panel was fabricated by 10 TE fibers, which were easily woven into a patch of cross-stitch, as shown in Figure 7i [13]. A strong signal was detected as opposed to the absence of signal for the non-contact ones when the finger touched TE fibers [13]. Based on this, Ding et al. accomplished hand-writing alphabetical “NUS”, and the output is shown in Figure 7h [13].

### 5.2. 3-Dimensional Generator

Generally, traditional film TE generators merely harvest the thermal energy in the in-plane direction without effectively utilizing the perpendicular temperature gradient between the human body and the environment [16,116]. Compared with a traditional structure, weaving TE fiber into three-dimensional structure fabric is adequate to reduce energy loss [16,111,116]. In Figure 7a, a textile structure was easily woven in a zigzag switch, and the density of TE couples was reduced due to the existence of insulating yarn. So, the power generation was as low as 0.11 Wm^−2^ per area at a temperature difference of 55 °C [111]. Additionally, a garter-stitch textile structure was designed without using insulating yarns. However, insulating yarns were utilized to enable better control of junction location during hand weaving, as illustrated in Figure 7b [111]. The garter-stitch textile provided output power per area and per couple for about 0.09 Wm^−2^ and 0.21 μW, respectively. Moreover, the tiger-yarn textile structure, a plain weave of identical segmented TE yarns with alternating segments of n- and p-type TE fibers, is shown in Figure 7c [111]. Additionally, the output power of tiger-yarn plain-weave is 0.62 Wm^−2^, which is higher than that of zigzag switch textile and garter-stitch textile. Improving on the previous study, Sun et al. designed a 3D thermoelectric generator without any supported substrates [116]. In Figure 7e, the π-type thermoelectric module was prepared by alternating doped carbon nanotube fibers, which were wrapped acrylic fibers [116]. This 3D thermoelectric generator was not only flexible but also stretchable by Utilizing elasticity originating from interlocked thermoelectric modules, as shown in Figure 7d [116].

### 5.3. Refrigeration

As the same application of thermoelectric materials, fiber-based TE materials can also be applied in solid-state cooling due to the Peltier cooling effect [5,8]. With the apparent advantages, such as reliability, flexibility, no moving parts, small volume, and precise temperature control, TE fibers are excellent candidates for cooling fabric to regulate human body thermal balance [8]. For example, Zhang et al. assembled a two-dimensional cooling textile using p-type Bi_0.5_Sb_1.5_Te_3_ fiber and n-type Bi_2_Se_3_ fiber, as shown in Figure 7f. With an input current of 2 mA, the cooling textile successfully achieved a maximum cooling of 5 °C.

## 6. Conclusions and Outlook

In recent years, developing a high TE performance and flexible thermoelectric fiber has attracted much attention. In this paper, we overview performances, device structures, and applications of state-of-the-art fiber-based thermoelectric materials. TE fibers can be divided into organic fibers, inorganic fibers (carbon fibers, semiconductor fibers, and oxide fibers), and hybrid fibers (carbon/organic fibers, semiconductor/organic fibers, and metal/organic fibers). As a material with excellent TE properties, inorganic TE fibers have limited applications due to their fragility, high cost, and complex preparation process. On the contrary, organic polymers have been studied extensively due to their high flexibility, low cost, and light weight. By combining inorganic materials and organic polymers, hybrid fibers have excellent TE properties and increased flexibility and have become promising material candidates. However, balancing flexibility and high TE performance to avoid the combination of defects of the two materials also faces some challenges. The reported preparations of TE fibers mainly include wet-spinning, electrospinning, thermal drawing, coating, etc. Furthermore, we summarize the temperature, Seebeck coefficient, electrical conductivity, thermal conductivity, power factor, ZT, and method of thermoelectric fibers according to the classification mentioned above in this paper, as shown in Table 1. Additionally, a great deal of work has been devoted to applying thermoelectric materials in generators, refrigeration, and sensors. As a one-dimensional structure, TE fibers cannot fully exploit their advantages by only connecting segmented TE fibers in series or parallel for application. A three-dimensional TE device fabricated by weaving or knitting with TE fibers can make full use of energy. For example, three-dimensional TE generators efficiently convert heat into electricity, using the temperature difference between the human body and ambience to the skin in a vertical direction. Different three-dimensional structures have further generated efficiencies. In conclusion, TE fibers show a broad application stage, which can be used in many fields in the future. The research on material, fabrication, and structure is continuously explored to find ideal TE fibers with high flexibility and excellent TE performance.

In short, fiber-based TE materials have had promising applications, but research is still in the early stages of development. Looking forward, we summarize the potential development of TE fibers and devices in the future; the following direction should be paid more attention.

### 6.1. Thermal Management

A flexible fiber thermoelectric device is an intelligent fabric that realizes a sizeable and flexible area by weaving with the industrial loom or cotton yarn, demonstrating certain cooling functions. This large-area thermoelectric fabric, which has flexible 3D structures, can be deformed, bent, and twisted and can cover curved surfaces with arbitrary geometry and an extensive temperature range. The refrigeration device based on thermoelectric fabric makes it possible to regulate human body temperature automatically and expands prospects for infrared stealth (thermal camouflage) in future military applications. Human body temperature regulation achieves fast temperature modulation by touching human skin with cooling elements, and thermal camouflage is accomplished by exposing thermoelectric materials to the outside and changing the surface temperature near the target. Both of these functions need efficient thermal management. In future research work, exploring a single high thermoelectric performance fiber with high mechanical properties for different temperature ranges is interesting and significant. Furthermore, the stability of thermoelectric and mechanical properties, as well as the comfort and practicality of wearing are also worth considering.

### 6.2. Medical Health and Exercise Monitoring

With the development of science and technology, people pay more and more attention to medical health. The advent of fabric electronics, including detectors composed of thermoelectric fibers, promises to break the time and space constraints of the traditional medical industry. At present, in medical treatment, the application of textile electronics is mainly in flexible measurement technology. Combining thermoelectric fiber with other sensors, such as strain, pressure, optical, gas, etc., and by monitoring some main physiological parameters of the human body, including body temperature, pulse, ECG, movement characteristics, and external environment, we can judge the current physiological state of the human body and provide early warning signals for sudden diseases or other high-risk conditions.

### 6.3. Wearable Power Generation and Integration

In addition, thermoelectric fiber can realize the mutual conversion of heat energy and electric energy, which has important applications in wearable power supplies for the human body. By developing thermoelectric fibers suitable for the range of human body temperature, a portable power supply can be realized, and the performance of thermoelectric fiber devices can be adjusted by optimizing device size, geometry, and weaving mode. In addition to being limited by its technical level, there are many challenges in applying fabric electronics. One challenge lies in reconciling the brittleness of thermoelectric materials with the flexibility of fibers: excellent thermoelectric properties means that the proportion of thermoelectric fibers is high, but the brittleness of thermoelectric materials is not conducive to flexibility. Another challenge is the need to make traditional hard materials flexible, washable, wearable, etc. In recent years, microelectronics and micro-electromechanical have developed rapidly, and the equipment is becoming more integrated and smaller. The integration of thermoelectric fiber power supplies, integrated circuits, and thin film electronic devices through material integration technology is also a consequent development direction of fabric electronics.

## Figures and Tables

**Table 1:** Different fiber types and their main properties.

Material	Temperature	Seebeck Coefficient (μVK^−1^)	Electrical Conductivity (Sm^−1^)	Thermal Conductivity (Wm^−1^K^−1^)	Power Factor (μWm^−1^K^−2^)	ZT	Method	Reference
Semiconductor TE Fiber								
Bi_2_Se_3_ core fibers	300 K	−150.85	31,900	1.25	725.9	0.18	thermal drawing technology	[28]
Bi_2_Te_3_ core fibers	300 K	130.50	74,400	0.52	1267.05	0.73	thermal drawing technology	[27]
Bi_2_Se_3_ nanowire	room temperature	−51	150,767	2.05	393.2	0.06	stress-induced method	[18]
Bi_0.5_Sb_1.5_Te_3_ core fibers	300 K	150	156,000	0.844	3520	1.25	thermal drawing technology	[29]
n-type Bi_2_Se_3_ core fibers	300 K	−92	77,000	0.839	651	0.23	thermal drawing technology	[29]
Sn–Se alloy core borosilicate glass-clad fibers	300 K	−151 (300 K)	550 (at 300 K) 6200 (at 900 K)	-	12.54 (300 K)	-	thermal drawing technology	[35]
single-crystal SnSe core fibers	862 K	~310	5500	0.22	528.55	1.94	thermal drawing technology	[38]
SnSe nanowires	370 K	~325	2200	0.55	232.38	0.156	vapor-liquid-solid (VLS) process	[19]
PbSnTe nanowires	300 K	33	141,414	1.3	~154	0.035	vapor-transport approach	[20]
PbTe Nanowires	room temperature	307	273	-	25.73	-	hydrothermal process	[43]
Ag_x_Te_y_-PbTe nanotubes	300 K	433 (30% of Ag)	3	-	0.567	-	electrospinning, electrodeposition and cation exchange reaction	[40]
glass fibers coated with PbTe nanocrystals	400 K	1542.4	172.4	0.226	410.16	0.73	solution-phase deposition method	[44]
Ag_2_Te nanowires film	420 K	−154.96	14,982	-	359.76	-	drop-coating methods	[24]
Carbon TE Fiber								
CNT-based fibers	room temperature	51.5	1740	-	4.61	-	electrostatic spray technique	[70]
PEI-doped CNT-based fibers	room temperature	−34.5	1655	-	1.97	-	electrostatic spray technique	[70]
carbon nanofiber-based cotton fibers	room temperature	−8	25.78	0.43	0.00165	1.15 × 10^−6^	dip-coated	[73]
lignin doped carbon nanotube yarns	room temperature	98.9	13,510	-	132.2	-	direct spinning method	[68]
F4TCNQ-doped CNT yarns	room temperature	81	248,100	-	1640 (room temperature) 2160 (400 K)	-	chemical vapor deposition	[74]
carbon nanotube threads	room temperature	48	48,000	-	11.06	-	enhanced direct injection pyrolytic synthesis (eDIPS) method	[71]
graphene fibers	290	–3.9	118,000	137	1.79	3.7 × 10^−6^	wet-spinning approach	[77]
bromine doped graphene fibers	350 K	~37.5	487,000	87	684.84	2.76 × 10^−3^	wet-spinning	[78]
graphene fibers	room temperature	22	1930	-	0.93	-	chemical reduction process	[17]
graphene fibers	room temperature	–15.6	950	-	0.23	-	chemical reduction process	[17]
oxide TE Fiber								
ZnO: Al-coated fibers	room temperature	–241.6	5.0	-	0.29	-	fibers coated method	[88]
La_0.95_Sr_0.05_CoO_3_ nanofibers	-	650	-	-	-	-	electrospinning	[86]
NaCo_2_O_4_ nanofibers	room temperature	128	-	-	-	-	electrospinning	[84]
Organic TE Fiber								
PEDOT: PSS fibers	room temperature	16.6	17,250	-	4.77	-	gelation process with a post-treatment	[94]
PEDOT: PSS–cotton fabric	-	10.3	30.6	0.0075	0.00325		impregnation	[95]
P3HT–cotton fabric	-	210	7	0.0068	0.309		impregnation	[95]
PCBM–cotton fabric	-	–283	1.33	0.0012	0.1066		impregnation	[95]
PEDOT: PSS fibers	-	19	83,000	-	30.0	-	wet-spun	[98]
PEDOT: PSS fibers	-	19.2	402,950	-	147.8	-	wet-spinning	[96]
PEDOT: PSS-coated CNF	room temperature	–58	87,100	-	293.0044	-	soaking method	[89]
Hybrid Fiber								
MWNTs/PVP fibers	300 K	−14	100	-	0.0196	-	coating	[103]
SWCNT/PVDF fibers	300 K	45.0	200,000	-	378		wet-spinning	[104]
n-type SWCNT/PVDF fibers	300 K	–39	185,000	-	289		wet-spinning	[104]
PEDOT: PSS /SWCNT fibers	room temperature	17.23	24,330	-	7.23	-	gelation process and ethylene glycol (EG) treatment.	[100]
PEDOT/CNT yarns	room temperature	48	67,900	-	157	-	dip-coating method	[75]
PEI-doped PEDOT/CNT yarns	room temperature	–23	56,100	-	29.68	-	dip-coating method	[75]
graphene and PEDOT: PSS hybrid fiber	room temperature	17.5	9630	0.16	2.9	-	hydrothermal process	[99]
PDMS/towel-gourd sponge (TS) fibers rGO–PPy composites	298.15 K	84.2	74	0.249	0.525	5.43 × 10^−4^	grafting method	[105]
PEDOT: PSS/tellurium nanowires	-	56	25,000	-	78.1	-	wet-spinning	[16]
PEDOT: PSS/tellurium nanowires	-	21.4	39,000	-	17.8	-	gelation	[107]
Ag nanowire/PEDOT: PSS-based coated yarn	300 K	0.8	32,000	-	0.0205	-	dip-coating process	[108]

## Data Availability

Not applicable.
